# Functional roles of antisense enhancer RNA for promoting prostate cancer progression

**DOI:** 10.7150/thno.51931

**Published:** 2021-01-01

**Authors:** Chun-Wu Pan, Simeng Wen, Lei Chen, Yulei Wei, Yuanjie Niu, Yu Zhao

**Affiliations:** 1 Department of Urology, Renji Hospital, School of Medicine, Shanghai Jiao Tong University, Shanghai, 200127, China.; 2 Department of Urology, The Second Hospital of Tianjin Medical University, Tianjin Medical University, Tianjin, 300211, China.; 3 Department of Gynecology and Obstetrics, Tianjin First Central Hospital, Tianjin, 300192, China.; 4 Department of Biochemistry and Molecular Biology, Mayo Clinic College of Medicine, Rochester, MN 55905, USA.

**Keywords:** Antisense eRNA, Enhancer RNA, Antisense RNA, Looping, DNA methylation

## Abstract

**Rationale:** Enhancer RNA (eRNA) bi-directionally expresses from enhancer region and sense eRNA regulates adjacent mRNA in cis and in trans. However, it has remained unclear whether antisense eRNAs in different direction are functional or merely a reflection of enhancer activation.

**Methods:** Strand-specific, ribosome-minus RNA sequencing (RNA-seq) were performed in AR positive prostate cancer cells. RNA-seq, GRO-seq, ChIP-seq, 4C-seq and DNA-methylation-seq that published in our and other labs were re-analyzed to define bi-directional enhancer RNA and DNA methylation regions. Molecular mechanisms were demonstrated by 3C, ChIP, ChIRP, CLIP, RT-PCR and western blot assays. The biological functions of antisense-eRNA were assessed using mice xenograft model and RT-PCR analysis in human tissues.

**Results:** In this study, we identified that antisense eRNA was regulated by androgen receptor (AR) activity in prostate cancer cells. Antisense eRNA negatively regulated antisense ncRNA in AR-related target genes' loci, through recruiting DNMT1 on the antisense enhancer in the gene-ending regions and elevating DNA methylation. Importantly, the chromatin exhibited a double looping manner that facilitated sense-eRNA to promoter and antisense-eRNA to gene-ending region in cis. Depletion of antisense eRNA impaired its neighbor mRNA expression, cancer growth and invasion. The expressions of antisense eRNA were correlated with biochemical recurrence and clinical marker *PSA*'s levels in patients' tissues.

**Conclusions:** The findings indicated that antisense eRNA was a functional RNA and may be a novel target that when suppressed improved prostate cancer therapy and diagnosis. New chromatin interaction among enhancer, promoter and gene-ending region might provide new insight into the spatiotemporal mechanism of the gene transcription and acting of bi-directional eRNAs.

## Introduction

Cells respond to changes in their environment through changes in tissue specific gene expression during development or in pathological changes 1, 2. Hormones, growth factors and neurotransmitters trigger programs of new gene expression temporally and spatially controlled by trans-acting transcription factors that binds to cis-acting DNA regulatory elements including promoters and enhancers 2, 3. Bidirectional non-coding RNAs (ncRNAs) are transcribed on enhancers, and are referred to as enhancer RNAs (eRNAs) 4-7. The observation is that eRNAs are produced from the enhancer region marked by histone H3 lysine 4 monomethylation (H3K4me1) and lysin 27 acetylation (H3K27ac) 4, 8. The activity of eRNAs increase the expression of not only neighboring but also intra-chromosomally distant genes, which transfer full enhancer activity to a or some target promoter(s) 6, 9-13. eRNA was reported to associate with the cohesin complex and facilitated estrogen receptor-α (ERα) induced enhancer and promoter looping 5, 7. Besides the looping, eRNA also enhance the RNA polymerase (Pol)-II elongation through removing the NELF and activating p-TEFb complex 14, 15. Thus, eRNA positively regulates neighboring messenger RNA (mRNA) expression in the same direction. The functional importance of the same-directional eRNA in regulated gene expression is well established. However, it has remained unclear whether antisense eRNAs in different direction are functional or merely a reflection of enhancer activation.

Antisense RNAs are unique DNA transcripts. Some of they are small, noncoding, and diffusible molecules, containing 19-23 nucleotides that complement mRNA 16-20. These small antisense RNAs included microRNA (miRNA) 21. Another particular group of ncRNAs are natural antisense transcripts (NATs) 16, 22, 23. They are widespread in eukaryotes and are transcribed in the opposite direction to protein coding transcripts. The first examples of bidirectional transcripts were detected as early as in the 1980s 24. These antisense transcripts have been found to originate from independent promoters, shared bidirectional promoters that are situated within genes. According to their orientation, they can be classified as head-to-head, tail-to-tail or internal. Most of bidirectional eRNAs are tail-to-tail manners 4. The antisense transcripts in gene coding region are complementary to a coding transcript from the opposite strand 16, 25. And then double-stranded RNA induces the degradation by Dicer system or inhibits splicing and translation 23, 26, 27. Moreover, nascent antisense transcript ANRIL1 recruits Polycomb repressive complex 2(PRC2) to inhibit transcription of CDKN2A 28. HOX transcript antisense RNA (HOTAIR) silences the homeoboxD (HOXD) locus in trans- through PRC2 recruitment 29. However, tail-to-tail bidirectional eRNAs have no complementary sequences and are not complementary to each other. The function of antisense eRNA remains still unestablished.

In this study, we identified sense eRNAs and antisense eRNAs were regulated by androgen receptor (AR) activity in prostate cancer cells. Antisense eRNA downregulated antisense ncRNA in *PSA*, *KLK2*, *FKBP5* and *TMPRSS2* loci, through recruiting DNMT1 on the antisense enhancer and enlarging DNA methylation in the gene-ending regions. Importantly, the chromatin exhibited a double looping manner that facilitated sense eRNA to promoter and antisense eRNA to gene-ending region in cis. Collectively, the findings in this study suggest that antisense eRNA was a functional RNA and may be novel target for cancer therapy and diagnosis. Accordingly, we reported a new interaction that enhancer, promoter and gene-ending region exhibited a spatiotemporally conformation acting mechanism through bi-directional eRNAs.

## Methods

### Cell lines, cell culture and reagents

Prostate cancer LNCaP cell lines were purchased from the American Type Culture Collection (ATCC). Prostate cancer C4-2 cell line was purchased from UroCorpoation. Cells were cultured in RPMI 1640 medium supplemented with 10% charcoal-stripped fetal bovine serum (FBS) or FBS (Invitrogen) (androgen-depleted medium) and 100 µg/ml penicillin-streptomycin-glutamine (Invitrogen) at 37°C with 5% CO_2_. For androgen stimulation experiments, LNCaP and C4-2 cells were grown in medium supplemented with charcoal-stripped serum for 48 h and then stimulated with 10 nM or 100 nM DHT (Sigma-Aldrich) for 24 h. For androgen receptor (AR) inhibition experiments, cells were grown with 10 µM or 20 µM enzalutamide (ENZ) (Sigma-Aldrich) for 24 h. siRNA control and siRNA for ERG were purchased from Dharmacon.

### Plasmids and antibodies

Flag-tagged DNMT1 WT and mutation were generated by cloning the corresponding cDNAs into pcDNA3.1 vector. *TMPRSS2-ERG* fusion gene (T1-E4) was generated by cloning the corresponding VCaP cDNAs into pcDNA3.1 vector. The cDNA fragments were amplified by Phusion polymerase (NEB) using Phusion High-Fidelity PCR Master Mix. PSA luciferase and ARE luciferase plasmids were described previously 15. The primers for cloning were shown in Supplementary Table 1. The insert and deletion mutants were constructed using KOD-plus-Mutagenesis Kit (TOYOBO, Japan). Antibodies: AR (Santa Cruz), DNMT1 (Abcam), DNA 5mC (Abcam), Flag (Sigma-Aldrich).

### Human prostate cancer specimens and RNA isolation from human tissues

Formalin-fixed paraffin-embedded (FFPE) or fresh hormone-naïve primary prostate cancer and castration resistant prostate cancer (CRPC) tissues were randomly selected from the Tianjin Medical Hospital and Shanghai Renji Hospital. Hormone-naïve patients with biopsy-proven prostate cancer have been treated at Shanghai Renji Hospital by radical retropubic prostatectomy between January 2005 and December 2016 without neoadjuvant therapy. 60 patients with CRPC were recorded the PSA levels every year. These samples with biochemical information were used for biochemical recurrence analysis and correlation analysis of *PSA* antisense eRNA and mRNA. 72 human single nucleotide polymorphisms (SNP) samples were used for RNA level measurement. The study was approved by the Tianjin Medical Hospital and Shanghai Renji Hospital Institutional Review Board (Ethical approval number: KY2019K036). FFPE tissues were collected and total RNAs were isolated using a RecoverAll Total Nucleic Acid Isolation Kit (Life Technologies). Isolation of RNAs from frozen human prostate cancer tissues was performed as described previously 30.

### RNA isolation from cultured cells, reverse transcription PCR (RT-PCR) and real-time PCR

RNA was extracted from tissues and cultured cells using TRIzol reagent (Invitrogen) or the RNeasy Plus Mini Kit (Qiagen) for human tissues according to the manufacturer's instructions. First-strand cDNA was synthesized with the PrimeScript Reverse Transcriptase Kit (Invitrogen). Reverse transcription and real-time PCR were performed as described previously 31. The PCR primers for genes are listed in Supplementary Table 1.

### Biotin-labeled RNA pull-down and western blot analysis

Biotin-labeled RNAs were *in vitro* transcript using Biotin RNA Labeling Mix (Roche) and T7 polymerase (New England Biolabs). C4-2 cells cultured in androgen-depleted medium were lysed in modified Binding buffer (150 mM NaCl, 50 mM Tris-HCl pH7.5, 1% NP-40, 0.1% SDS and 1% protease inhibitor cocktails). Cell lysates were incubated with biotin-labelled RNAs and streptavidin beads at 4ºC for 12 h. The beads were washed in wash buffer (50 mM Tris, pH 7.4; 150 mM NaCl; 0.05% Nonidet P-40 (NP-40); 1 mM MgCl_2_) at 4°C six times. The samples were subjected to western blot analyses as described previously 31. Briefly, protein samples were denatured and subjected to SDS-polyacrylamide gel electrophoresis (SDS/PAGE), and were transferred to nitrocellulose membranes (Bio-Rad). The membranes were immunoblotted with specific primary antibodies, horseradish peroxidase-conjugated secondary antibodies, and visualized by SuperSignal West Pico Stable Peroxide Solution (Thermo Fisher).

### Chromatin immunoprecipitation (ChIP) and methylated DNA immunoprecipitation (MeDIP)

ChIP was performed following our previous protocol as described 15, 32. MeDIP was performed following previous protocol as described 33. The qPCR analysis was performed using the primers listed in Supplementary Table 1.

### RNA-seq and data analysis

Total RNA was extracted from LNCaP and C4-2 cells cultured in androgen-depleted media for 72 h or ENZ treatment for 48 h. RNAs were harvested using the miRNeasy kit (Qiagen) and RNA quality was assessed using an Agilent Bioanalyzer. High quality (Agilent Bioanalyzer RIN >7.0) total RNAs were employed for the preparation of sequencing libraries using Illumina TruSeq Stranded Total RNA/Ribo-Zero Sample Prep Kit. A total of 500-1,000 ng of riboRNA-depleted total RNA was fragmented by RNase III treatment at 37°C for 15 min and RNase III was inactivated at 65°C for 10 min. Size selection (50 to 150 bp fragments) was performed using the FlashPAGE denaturing PAGE-fractionator (Life Technologies) prior to ethanol precipitation overnight. The resulting RNA was directionally ligated, reverse-transcribed and RNase H treated.

Samples with biological duplicates were sequenced using the Illumina HiSeq2000 platform at the Mayo Genome Core Facility. Pre-analysis quality control was performed using FastQC (http://www.bioinformatics.babraham.ac.uk/projects/fastqc/) and RSeQC software 34 to ensure that raw data are in excellent condition and suitable for downstream analyses. Pair-end raw reads were aligned to the human reference genome (GRch37/hg19) using Tophat 35. Genome-wide coverage signals were represented in BigWig format to facilitate convenient visualization using the UCSC genome browser. Gene expression was measured using RPKM (Reads Per Kilo-base exon per Million mapped reads) as described previously 36. Correlation analyses between eRNA and mRNA expression were performed using Python and R scripts. EdgeR 37 was used to identify genes that were differentially expressed between CRPC and primary prostate tumors. Raw and processed data have been analyzed from NCBI Gene Expression Omnibus with accession number GSE55032.

### Cross-linking immunoprecipitation (CLIP)

5 × 10^6^ C4-2 cells treated with 100 µM 4-Thiouridine (4SU) for 8 h were washed with cold PBS one time and cells were irradiated once with 150 mJ/cm^2^ at 365 nm using a Startalinker 38. Cells were lysed in lysis buffer (50 mM Tris-HCL, pH 7.4, 100 mM NaCl, 1% NP-40, 0.1% SDS, 0.5% sodium deoxycholate, protease inhibitor cooktail and RNase inhibitors) with protease inhibitors (1 mL) and transferred to 1.5 mL microtubes. Lysate was partially digested by 1 U/µL RNaseT1/A for 15 min at 22°C. RNA was immunoprecipitated with DNMT1 or Flag antibodies and protein A/G beads for 16 h at 4°C. After washed for 6 times, RNA was phosphorylated by T4 PNK and ligated RNA between 3' and 5' ends by RNA T4 ligase. SDS-PAGE loading buffer was added and the mixture was incubated at 70°C for 10 min. After running the SDS-PAGE gel, the RNA-protein complexes were transferred from gel to a nitrocellulose membrane using a wet transfer apparatus (30 V for 1 h). The membrane with target protein was cut up, and the targeted membrane piece was incubated with Proteinase K for de-crosslink. After de-crosslink, RNA was reverse transcribed into cDNA and subjected to real-time qPCR analysis.

### Quantitative real-time Reverse Transcription polymerase chain reaction (qRT-PCR) analysis

Relative RNA levels determined by qRT-PCR were measured on a Bio-Rad CFX96 Real-Time PCR System, using Taqman Mix (Invitrogen) or SYBG Mix (BioRad). Different probes were used to distinguish the sense and antisense RNAs. All primers were obtained from Rebio Pharmaceuticals (Guangzhou, China), and gene-specific sequences-probes are listed in Supplementary Table 1. The relative expression of RNAs was calculated using the -ΔΔCt method 15, 39. *GAPDH* was used as an internal control for quantification of gene targets.

### Locked nucleic acids (LNA)-mediated knockdown

LNAs targeting antisense RNAs were designed and obtained from Rebio Pharmaceuticals (Guangzhou, China). Transfections with LNAs (50 µM) were performed with Lipofectamine RNAiMAX (Thermofisher) or siRNAs were performed with Lipofectamine 2000 (Invitrogen) according to the manufacturer's instructions. RNAs and protein were harvested for analysis 72 h after transfection.

### Chromatin isolation by RNA purification (ChIRP)

The ChIRP experiment was performed essentially following the original protocol as described previously 15. The online Biosearch Technologies' Stellaris FISH Probe Designer was used to design antisense oligo probes tiling PSA and FKBP5 antisense eRNA. The probe oligos were synthesized with a 3'-Biotin-TEG modification and purified by HPLC. The ChIRP probes are shown in Table S1.

### Design and screening of antisense oligonucleotides (ASOs)

The ASOs were designed using a full phosphorothioate backbone and a 10-base 2′-deoxynucleoside gap flanked by 2′-O-methyl (cMt)-modified nucleotides. The motif for the ASOs targeting the eRNAs tested was mmm-10-mmm, where m represents cMt modification and -10- represents the 10-base DNA gap. ASOs were synthesized and purified as described previously 40. A large number of ASOs were screened by BGI Genomics Inc. (Shenzhen, China) for high efficient reduction of RNA.

### Mouse xenograft generation and tumor growth measurement

The mouse study was approved by Tianjin Medical University Institutional Animal Care and Use Committee. Six-week- old NSG male mice were injected with 5 × 10^6^ of cancer cells infected with lentivirus or shRNAs and/or expression vectors in 100 μl PBS with 100 μl of Matrigel matrix (BD Bioscience) in right flanks. After the tumor became about 100 mm^3^, the 100 mg/kg ASOs were injected by intraperitoneal (IP) every week. Tumors were monitored until they reach maximum tumor volumes of 1,000 mm^3^ and tumor growth was measured with caliper every 3 days.

### Western blotting

Prostate cancer cells were harvested and lysed by RIPA buffer on ice, the supernatant was quantified by BCA protein quantification assay (Bio-Rad, USA). Equal amounts of protein sample were added to 4× sample buffer and boiled for 10 min. The sample was subjected to SDS-PAGE analysis and transferred to nitrocellulose membrane. The membrane was blocked by 5% milk for 2 h at room temperature and incubated with primary antibody at 4°C overnight. The second day, the membrane was washed three times with 1 × TBST and incubated with horseradish peroxidase-conjugated secondary antibodies for 1 h at room temperature. The protein bands were visualized by super signal West Pico Stable Peroxide Solution (Thermo Fisher Scientific, USA).

### Quantitative chromosome conformation capture (3C) assay

3C assays were performed following our protocol as described previously 15. Briefly, the cross-linked chromatin was digested with specific restriction enzymes overnight. The crosslinking was reversed and ligated DNA was purified. The qPCR analyses need additionally 3% DMSO in the final buffer. The qPCR analysis was performed using the primers listed in Supplementary Table 1.

### MTT assay and colony formation assay

3-(4,5-dimethylthiazol-2-yl)-2,5-diphenyltetrazolium bromide (MTT) was used for MTT assay as previously description 41, 42. Briefly, 500 cells were plated into each well of 6-well plate. Approximately 14 days later, the colonies were fixed with methanol: acetic acid (7:1) for 30 min in room temperature and stained with (0.5% w/v) trypan blue for 1 h. The colonies with more than 50 cells were counted.

### Invasion assay

Cell invasion assay *in vitro* was performed with Corning matrigel invasion chamber assay according to manufacturer's instructions as previously described 38. Briefly, cells were diluted to 3 × 10^4^ cells per well in serum-free medium and plated to the inside of matrigel chamber in 24-well plates, outside the chamber was added the medium with 10% FBS. After 24 h, cells were fixed in methanol for 20 min and then stained with 1 mg/ml crystal violet staining for 30 min. The membranes of chamber were covered by coverslip and observed using microscope after 6 times washing. Wells were repeated in triplicate, and the invaded cells were quantified per field of view. Eight fields of three independent replicates were recorded and analyzed.

### Statistical analysis

Experiments were carried out with three or more replicates unless otherwise stated. Statistical analyses were performed using ANOVA test and Student *t* test for most comparisons. *P* < 0.05 is considered statistically significant. Non-parametric Kolmogorov-Smirnov (KS) test was used to evaluate statistical significance of differential expression between primary prostate cancers.

## Results

### Antisense eRNA downregulates antisense ncRNA on AR target loci

**Figure 1:**
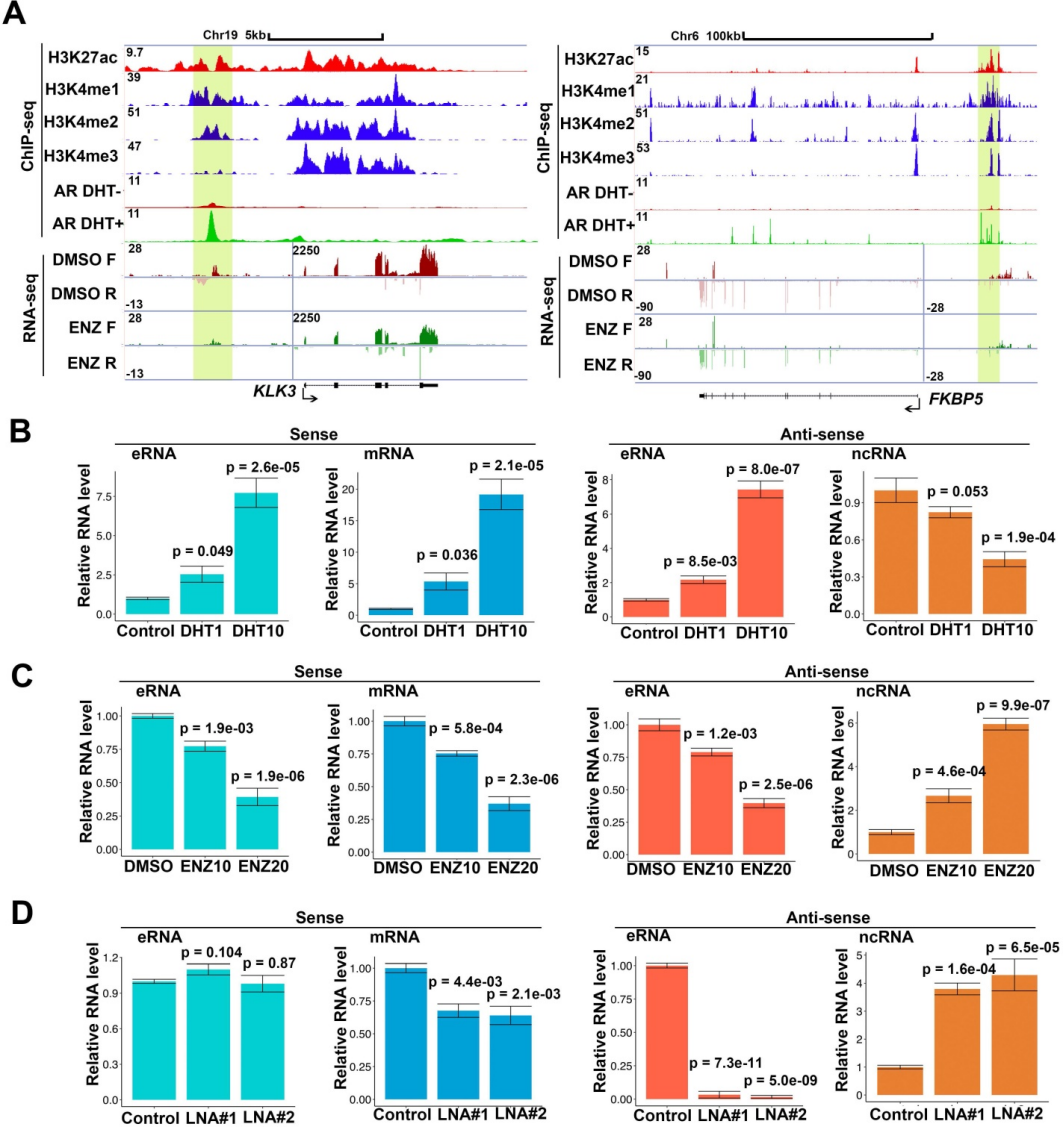
** Antisense eRNA is negatively related with antisense mRNA on AR target loci. (A)** Screen shots from UCSC genome browser showing signal profiles of eRNA and mRNA expression in LNCaP. ChIP-seq in LNCaP and C4-2 were shown as a reference. *PSA (KLK3)* was in left panel; *FKBP5* was in right panel. The enhancer regions were highlighted in yellow box. **(B)** Sense eRNA, sense mRNA, antisense eRNA (as-eRNA) and antisense ncRNA expressions of *PSA* were measured by qRT-PCR in C4-2 cells treated with DHT. Means and standard deviations (error bar) were determined from three replicates. Error bars represented mean ± SD for triplicate experiments. *P* values were shown in the figures. *GAPDH* as internal control. **(C)** Sense eRNA, sense mRNA, antisense eRNA (as-eRNA) and antisense ncRNA expressions of *PSA* were measured by qRT-PCR in C4-2 cells treated with enzalutamide (ENZ). Means and standard deviations (error bar) were determined from three replicates. Error bars represented mean ± SD for triplicate experiments. *P* values were shown in the figures. *GAPDH* as internal control. **(D)** Sense eRNA, sense mRNA, antisense eRNA (as-eRNA) and antisense ncRNA expressions of *PSA* were measured by qRT-PCR in C4-2 cells knocked down with antisense eRNA LNAs. Means and standard deviations (error bar) were determined from three replicates. Error bars represented mean ± SD for triplicate experiments. *P* values were shown in the figures. *GAPDH* as internal control.

Notably, RNA polymerase II (RNAPII) at enhancers transcribes bi-directionally the class of eRNAs within enhancer region and eRNAs also express in bi-directional in the enhancer region 4, 7, 8. To further study the eRNAs expressed profiles, we chose androgen stimulated androgen receptor (AR) and AR-related eRNA as model systems. To further study the different direction of transcripts, we performed strand-specific, ribosome-minus RNA sequencing (RNA-seq) in AR positive prostate cancer cells. In parallel, we used AR, H3K4me1 3, 43, H3K4me2 3, H3K4me3 3 and H3K27ac 44 chromatin immunoprecipitation and sequencing (ChIP-seq) to define the AR related enhancer and promoter region in genome. We totally found 6,001 AR related eRNAs in AR-bound enhancer in C4-2 cells and approximated 30% of eRNAs were bi-directionally observed by strand-specific RNA-seq (Figure S1A and top 600 (FDR<0.001) eRNAs in Table S2). AR related eRNAs can be upregulated by androgen (dihydrotestosterone (DHT)) or impaired by enzalutamide (ENZ) 42, 45-47. However, it remains unclear whether both directional eRNAs can be regulated by AR activity. A meta-analysis of both global nuclear run-on sequencing (GRO-seq) 8 and our strand-specific RNA-seq indicated that bi-directional *PSA* eRNAs (sense eRNAs and antisense eRNAs) were upregulated by DHT in GRO-seq data (Figure S1B) and were deterred by ENZ in RNA-seq data (Figure 1A). Furthermore, we found that bi-directional eRNAs simultaneously be regulated by ENZ in *FKBP5, KLK2* and *TMPRSS2* loci (Figure 1A and Figure S1C). Based on the GRO-seq and RNA-seq in LNCaP cells 8, 15, 48, we observed that antisense ncRNA also expressed in *PSA, FKBP5, KLK2 and TMPRSS2* gene coding region (Figure 1A, S1A and S1C) which were more than 10-fold lower than sense transcripts as previous reported 49. However, the internal control *ACTB* and *GAPDH* failed to be regulated by DHT or ENZ in RNA-seq and RT-PCR data (Figure S1D and S1E), which ruled out the possibility that levels of antisense eRNAs and ncRNAs were changed because of different RNA inputs. To confirm these observations, we performed the bi-directional eRNAs, antisense ncRNAs and mRNAs' expressions in C4-2 cells by RT-qPCR. Consistent with GRO-seq and RNA-seq, we found that bi-directional eRNAs and mRNAs were increase with DHT treatment in dose-dependent manner (Figure 1B and S1F). Interestingly, antisense ncRNAs in *PSA* gene coding region were decrease with DHT treatment in dose-dependent manner (Figure 1B). Consistently, bi-directional eRNAs and mRNAs were downregulated by AR antagonist ENZ and antisense ncRNAs were upregulated by ENZ in dose-dependent manner (Figure 1C and S1F). To further search the antisense eRNA's function, we knocked down *PSA* antisense eRNA by siRNA mixture pool. The data showed that sense eRNA was no significant change with antisense eRNA knocking down (KD) (Figure 1D). However, *PSA* mRNA was downregulated and *PSA* antisense ncRNA was upregulated by antisense eRNA KD (Figure 1D). Sense eRNA mediated the same directional mRNA through affecting enhancer-promoter looping and RNA polymerase II (Pol II) ser2 phosphorylation 14, 15. Our data showed that *PSA* antisense eRNA KD had no effect in impairing enhancer-promoter looping and Pol II phosphorylation (Figure S1G). Using specific single-strand locked nucleic acids (LNA) to block antisense ncRNA of *PSA* indicated that antisense eRNA failed to mediate *PSA* mRNA without *PSA* antisense ncRNA expression (Figure S1H). Taken together, these data suggest that *PSA* antisense eRNA mediated mRNA through inhibiting antisense ncRNA at *PSA* gene locus.

### Antisense eRNAs increase DNA methylation in the gene-ending region to block antisense ncRNA transcript

**Figure 2:**
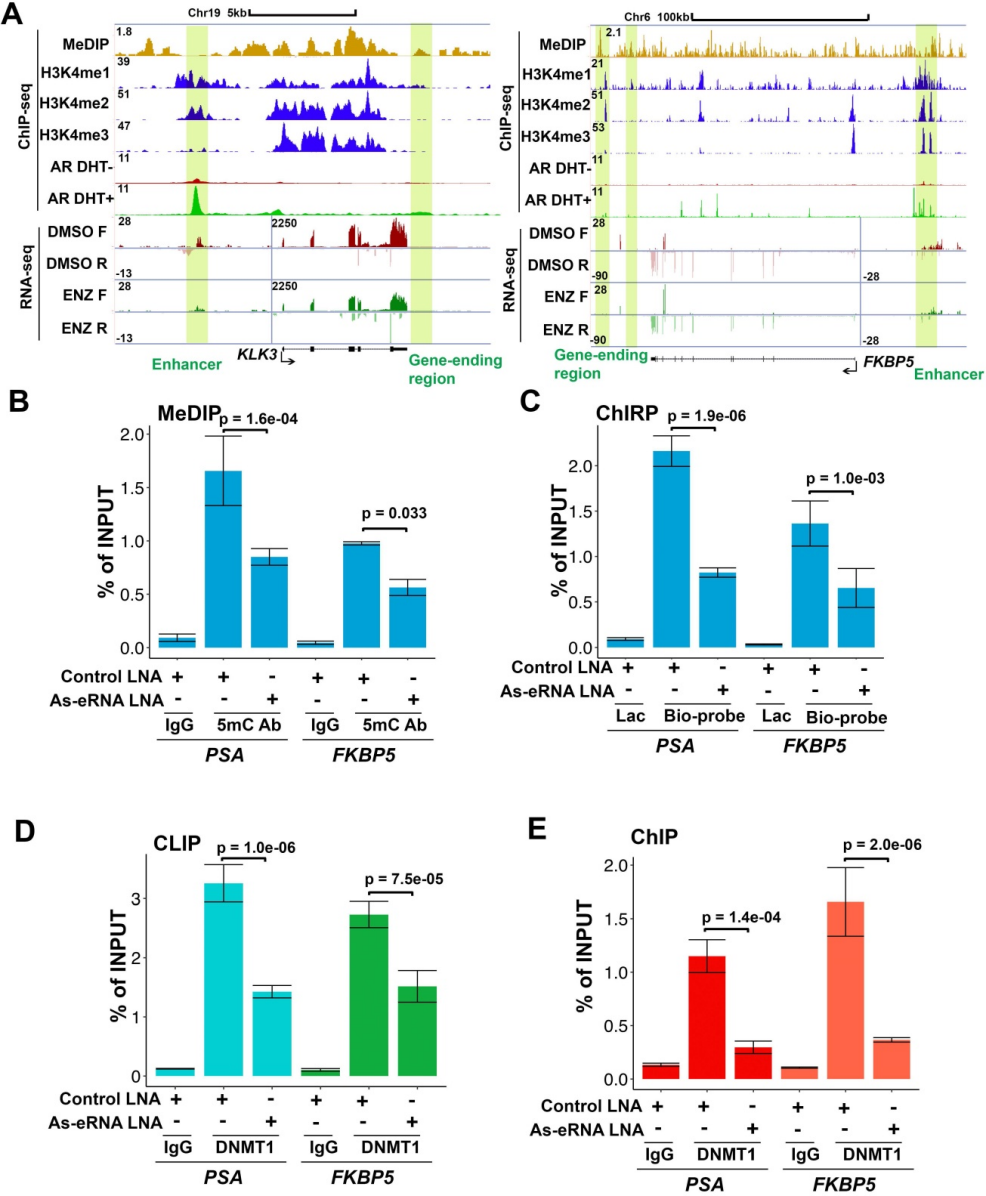
** DNA methylation in antisense promoter is critical for expression of antisense ncRNA. (A)** Screen shots from UCSC genome browser showing signal profiles of eRNA and mRNA expression in LNCaP and C4-2. MeDIP-seq in Abl cells are shown as a reference. *PSA* is in left panel; *FKBP5* is in right panel. The enhancer regions are highlighted in yellow box. **(B)** Quantitative PCR verification of MeDIP results on the antisense enhancer (gene-ending region) of *KLK3 and FKBP5* with DNA 5mC antibody against the 5mC sites in C4-2 cells. Means and standard deviations (error bar) were determined from three replicates. Error bars represented mean ± SD for triplicate experiments. *P* values were shown in the figures. **(C)** ChIRP assay using biotin-labeled LacZ or *antisense eRNA (KLK3 or FKBP5)*-specific DNA probes and streptavidin beads. Pull-down DNA was analyzed by real-time PCR. All data shown were mean values ± SD (error bar) from three replicates. *P* values were shown in the figures. **(D)** CLIP-qPCR analysis of DNMT1 binding at the *KLK3 and FKBP5 antisense RNA* in C4-2 cells transfected with control or antisense-specific LNAs. Immunoprecipitated RNAs were detected by real-time PCR. All data shown were mean values ± SD (error bar) from three replicates. *P* values were shown in the figures. **(E)** ChIP-qPCR analysis of DNMT1 binding at the *KLK3 and FKBP5* gene-ending region in C4-2 cells transfected with control or antisense-specific LNAs. Immunoprecipitated DNAs were detected by real-time PCR. All data shown were mean values ± SD (error bar) from three replicates. *P* values were shown in the figures.

Because *PSA* antisense eRNA KD had no effect in impairing enhancer-promoter looping and RNA Pol II phosphorylation in the *PSA* locus, there should be another molecular mechanism that *PSA* antisense eRNA regulated the antisense ncRNA. To study the new mechanism for antisense ncRNA regulating, we analyzed the methylated DNA immunoprecipitation sequencing (MeDIP-seq) in LNCaP and LNCaP-abl cells 33. The AR activity is higher in castration resistant LNCaP-abl cell line than hormone-naïve line LNCaP cells. We found that 5-methylcytosine (5 mC) levels were increase in the ending of gene body region of top 100 eRNA-adjacent mRNAs in LNCaP-abl cells compared to LNCaP cells (Figure S2A). It is an interesting phenomenon because high level of 5mC in promoter and enhancer means inhibition of the related neighbor gene 33, 50, 51. Thus, we hypothesized that the gene-ending region may be the promoter or enhancer region of antisense ncRNA (Figure 2A, yellow box). To test our hypothesis, we searched the enhancer and promoter makers in the gene-ending region through ChIP-seq data. Interestingly we found that there were enhancer makers H3K4me1 in the gene-ending regions of *PSA, FKBP5, KLK2* and* TMPRSS2* loci, and also androgen-stimulated AR binding in the same loci (Figure 2A and S2B). Importantly, there were significant 5mC peaks overlapped with H3K4me1 and AR peaks in the gene-ending regions of *PSA, FKBP5, KLK2* and* TMPRSS2* loci (Figure 2A and S2B), suggesting that DNA methylation play a critical role for antisense ncRNA. Next, we confirmed that DNA methylation were downregulated with antisense eRNA KD by LNA in *PSA* and *FKBP5* loci (Figure 2B). Furthermore, ChIRP assay demonstrated that antisense eRNA KD impaired the RNA binding to gene-ending regions of *PSA* and *FKBP5* loci (Figure 2C). It has been reported that DNMT1 interacts with ncRNA to increase 5mC in specific genes' promoters 52, 53. To verify the interaction between DNMT1 and antisense eRNAs, we performed CLIP assay to demonstrate them for *PSA* and *FKBP5* antisense eRNAs. CLIP assay showed that *PSA* and *FKBP5* antisense eRNAs associated with DNMT1, and KD of *PSA* and* FKBP5* antisense eRNAs decreased the interaction between DNMT1 and antisense eRNAs (Figure 2D). Furthermore, KD of *PSA* and* FKBP5* antisense eRNAs downregulated the DNMT1 binding to gene-ending region of *PSA* and *FKBP5* loci (Figure 2E). These data suggest that antisense eRNAs recruited DNMT1 to gene-ending region of *PSA* and *FKBP5* and increased 5mC levels on *PSA* and *FKBP5* loci.

### Antisense eRNAs interacts with DNMT1 through specific secondary structure

**Figure 3:**
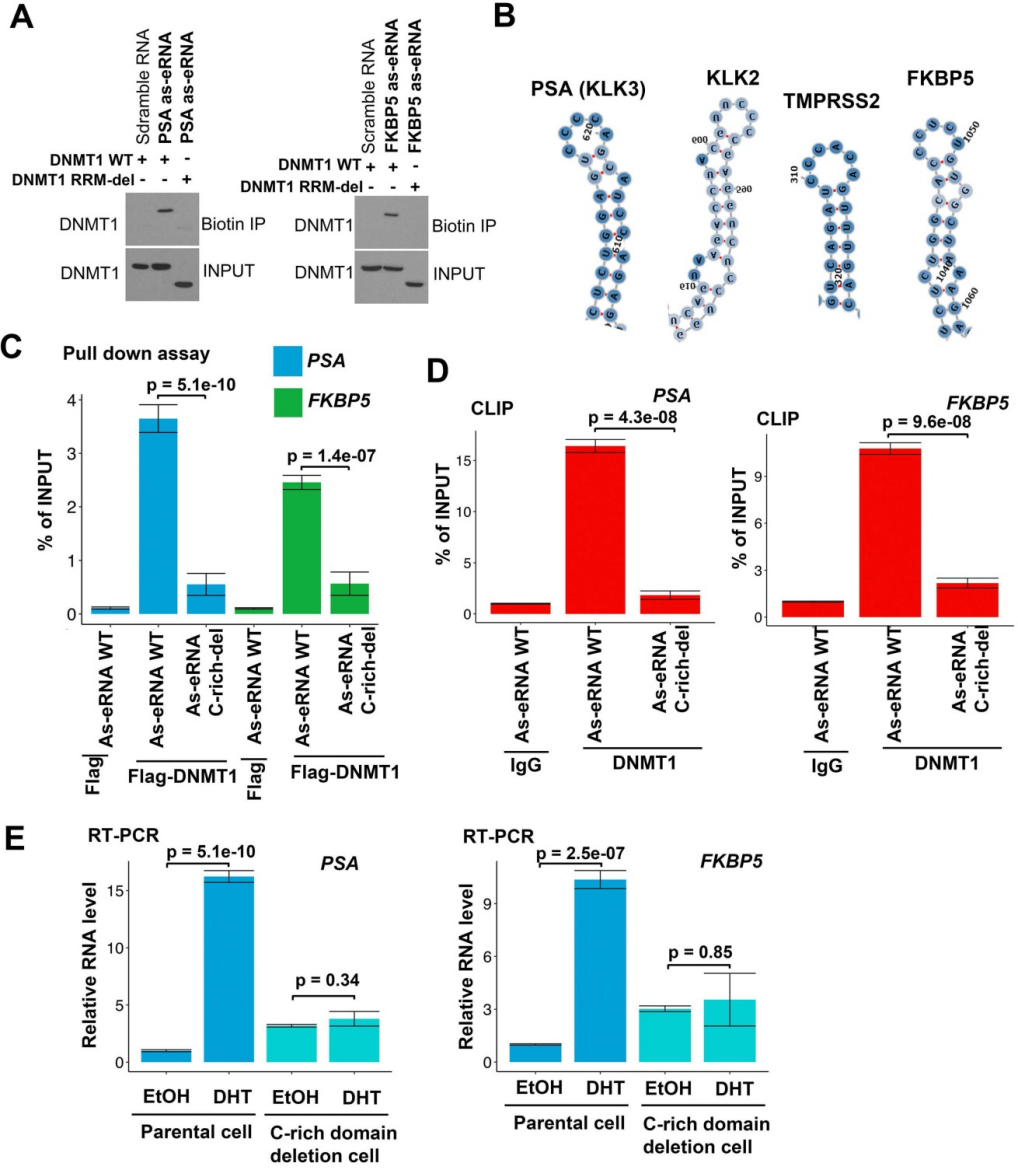
** DNMT1 binds to antisense eRNA through secondary structure. (A)** Biotin pull‐down assay by incubating biotin‐labeled specific probes targeting *PSA* and *FKBP5* antisense eRNAs (as-eRNAs) with C4-2 cell lysate followed by Western blot with DNMT1 antibodies. **(B)** Secondary structure of *PSA, KLK2, TMPRSS2 and FKBP5* predicted by https://rna.tbi.univie.ac.at. **(C)** Flag-DNMT1 pull-down antisense eRNA *in vitro*. Flag-DNMT1 was produced using Quick coupled transcription/translation kit through T7 promoter *in vitro*. Antisense RNAs was transcribed by T7 polymerase *in vitro*. The purified proteins were pulled down by Flag antibodies and tested by western blot. **(D)** CLIP-qPCR analysis of DNMT1 binding at the *KLK3 and FKBP5* antisense eRNA (as-eRNAs) in C4-2 cells transfected with WT or mutated antisense eRNAs. Immunoprecipitated RNAs were detected by real-time PCR using primers targeting plasmids to rule out endogenous RNA. All data shown were mean values ± SD (error bar) from three replicates. *P* values were shown in the figures. **(E)** The expressions of PSA and FKBP5 are measured by qRT-PCR in C4-2 parental cells and CRISPR-generated antisense eRNA C-rich domain-deletion cells treated with or without DHT. Means and standard deviations (error bar) were determined from three replicates. Error bars represented mean ± SD for triplicate experiments. *P* values were shown in the figures. *GAPDH* as internal control.

DNMT1 binds to ncRNA through its RNA recognized motif (RRM) 54. To verify whether antisense eRNAs bound to DNMT1 through the same region, we generated the RRM deletion mutant of DNMT1 plasmid. Biotin labeled RNA pull-down assay showed that DNMT1 wild type (WT) was able to interact with *PSA* and *FKBP5* antisense eRNAs, but RRM deletion mutant failed to bind to antisense eRNAs (Figure 3A). These data indicated that DNMT1 bound to antisense eRNAs through the same RRM. To further understand the detailed domain of antisense eRNA being responsible for binding to DNMT1, we predicted the four secondary structures of *PSA, FKBP5, KLK2* and *TMPRSS2*. We found that there was Cytosine (C)-rich stem loop in all these antisense eRNAs (Figure S3A-D and 3B). To test the function of C-rich region, we transcript *PSA* and *FKBP5* WT and C-rich region deleted antisense eRNAs *in vitro* and transcript/translated Flag-DNMT1 through T7 promoter *in vitro*. *In vitro* Flag pull-down assay showed that Flag-DNMT1 bound to *PSA* and *FKBP5* WT antisense eRNAs, but not C-rich domain deleted antisense eRNAs (Figure 3C). Furthermore, to test whether interaction between DNMT1 and antisense eRNA relies on C-rich domain, we overexpressed WT and C-rich-deletion mutant antisense eRNA in C4-2 cells and measured them using primers on plasmids to rule out the endogenous RNA contamination. CLIP assay showed that C-rich domain-deletion antisense eRNAs lose about 90% interaction with DNMT1 compared to antisense eRNAs WT in C4-2 cells (Figure 3D). To measure the effect of C-rich domain on antisense eRNAs and mRNA expression, we overexpressed *PSA* and *FKBP5* antisense eRNAs in C4-2 cells with or without C-rich region-deletion in genome by CRISPR system (Figure S3E). DHT upregulated *PSA* or *FKBP5* mRNA levels in C4-2 cells, but DHT failed to increase mRNAs in C-rich domain deletion cells (Figure 3E). These data indicated that C-rich domain was the key domain for antisense eRNA binding to DNMT1 and antisense eRNA's function.

### Two-looping interaction facilities the spatial targeting of antisense eRNAs to gene-ending region

**Figure 4:**
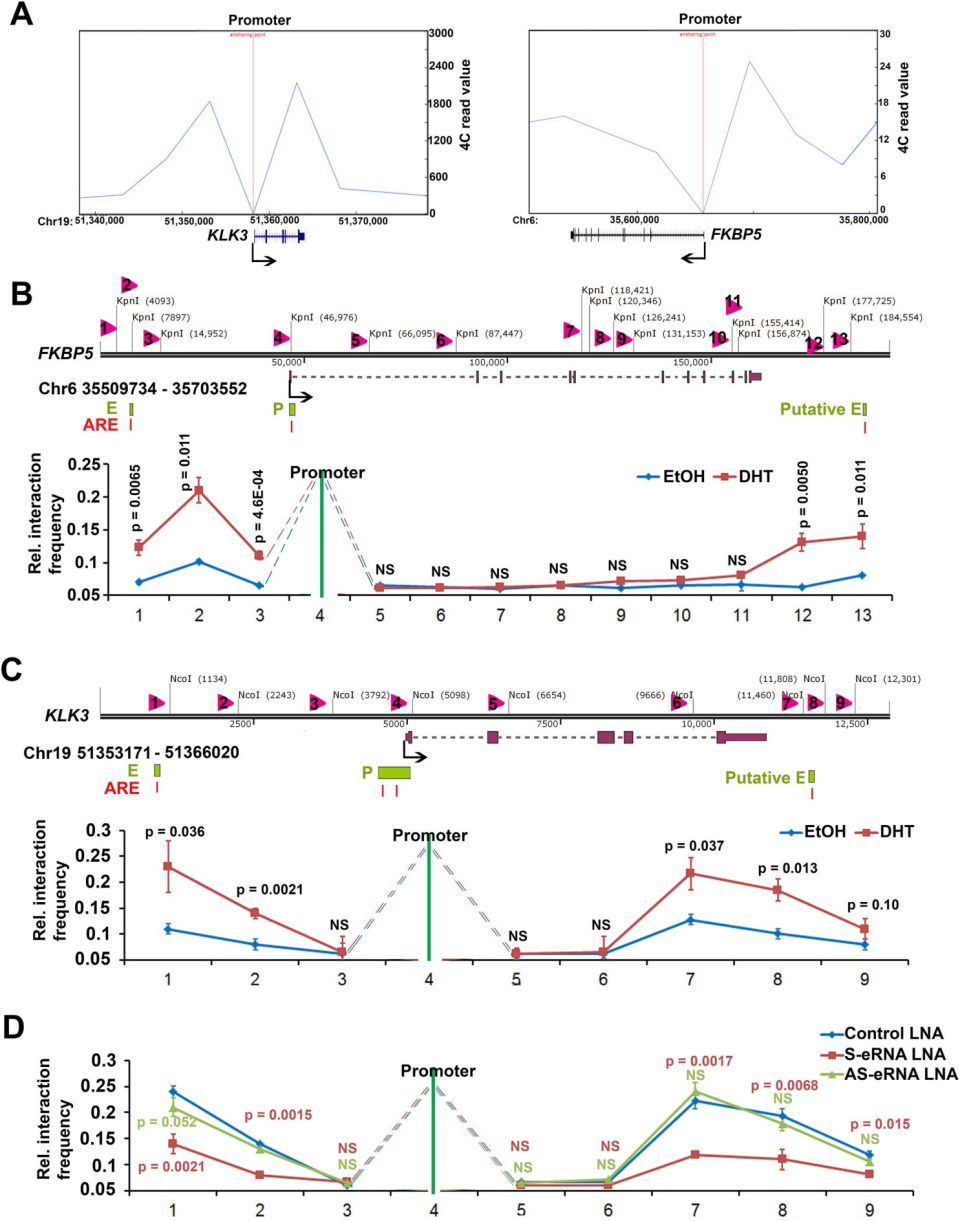
** Antisense eRNA's function in cis relies on new two-looping interaction. (A)** Virtual circularized chromosome conformation capture (4C)-seq data from http://promoter.bx.psu.edu/hi-c/chiapet.php showing potential signal binding regions with *PSA* and* FKBP5* promoter in NHEK GM12878 and LNCaP cells. **(B and C)** The diagram showed that chromosome conformation capture (3C) assay with NcoI digesting in PSA locus and KpnI digesting in FKBP5 locus. Evaluation of the enhancer-promoter-antisense enhancer (gene-ending region) interaction at *FKBP5* and *PSA* target gene loci were showed by chromosome conformation capture (3C) assays. Input meant that the genomic DNA was not crossed link with protein, then was digested by NcoI (for *PSA*) or KpnI (for *FKBP5*) and ligated randomly. Input DNA meant genomic random ligated DNA. Error bars represented mean ± SD for triplicate experiments (n=3). *P* values were shown in the figures. NS, no significance. **(D)** Evaluation of the enhancer-promoter-antisense enhancer (gene-ending region) interaction at *PSA* target gene loci by 3C assays in sense eRNA (S-eRNA) or antisense eRNA (As-eRNA) knocked down by LNAs. Error bars represented mean ± SD for triplicate experiments (n=3). *P* values were shown in the figures. NS, no significance.

Sense eRNAs mainly mediate adjacent mRNA in cis 5, 6. The question is how antisense eRNAs recognize and play a role in cis. To solve this question, we analyzed the genome spatial interaction meta-data published previously 55-57. Circularized chromatin conformation capture (4C)-seq data in AR-positive GM12878 and LNCaP cells showed that the promoters of *PSA, FKBP5, KLK2* and *TMPRSS*2 had potential interacted peaks with the enhancer regions and also with gene-ending regions related to promoters (Figure 4A and S4A). To confirm these chromatin conformation interactions, we completed chromatin conformation capture (3C) assay in C4-2 cells. We found that the digest sites near the enhancer regions and gene-ending regions interaction with promoters were stimulated by DHT in C4-2 cells in *PSA*, *FKBP5*, *KLK2* and *TMPRSS2* loci (Figure 4B, 4C, S4B and S4C). These data indicated that there were two interacted sites near the promoter of these AR target genes. eRNAs play the important role in chromatin interaction 5, 15, 58, 59. To test the functional roles of sense eRNAs and antisense eRNAs in chromatin interaction, we knocked down them by LNAs in C4-2 cells. We found that KD of sense eRNAs impaired enhancer-promoter interaction and promoter-gene-ending interaction in *PSA* locus (Figure 4D). However, KD of antisense eRNA did not affect these chromatin interaction (Figure 4D). We concluded that antisense eRNA mediated mRNA through binding to DNMT1, but not chromatin interaction. Promoter-gene-ending interaction increased the specific location of antisense eRNA targeting to gene-ending region.

### Antisense eRNAs and antisense-enhancers mediate tumor growth and *PSA* levels in cells and in tissues

**Figure 5:**
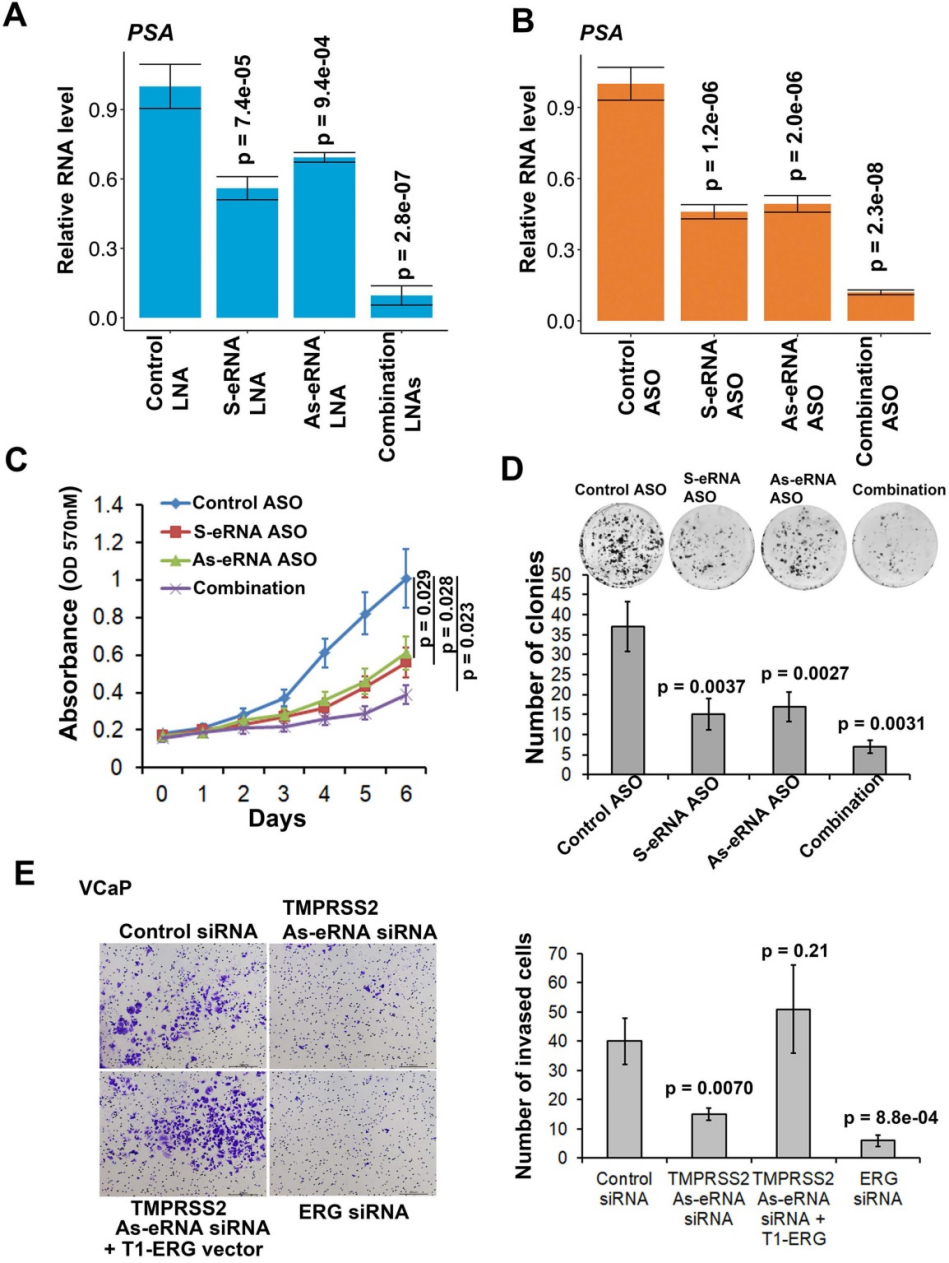
** Antisense eRNA and antisense-enhancer mediate mRNA in cells. (A and B)** The expressions of *PSA* are measured by qRT-PCR in C4-2 cells transfected by LNAs (A) or ASOs (B). Means and standard deviations (error bar) were determined from three replicates. Error bars represented mean ± SD for triplicate experiments (n=3). *P* values were shown in the figures. *GAPDH* as internal control. **(C)** C4-2 cells were transfected with control ASO, PSA sense eRNA (S-eRNA) ASO, PSA antisense eRNA (As-eRNA) ASO, or S-eRNA and As-eRNA combination (combination) using lipofectamine (2000 cells for each well). Cell viability conditions were determined by MTT assay. Error bars represented mean ± SD for triplicate experiments (n=6). *P* values were shown in the figures. **(D)** C4-2 cells were transfected with control ASO, PSA sense eRNA (S-eRNA) ASO, PSA antisense eRNA (As-eRNA) ASO, or S-eRNA and As-eRNA combination (combination) using lipofectamine (500 cells for each well). After 14 days, cells were fixed and stained by typen blue. Colonies (>50 cells) were calculated for each well. Error bars represented mean ± SD for triplicate experiments (n=3). *P* values were shown in the figures. **(E)** 3×10^4^ VCaP cells were cultured in each well of 24-well transwell plate. VCaP cells were transfected with control siRNA, TMPRSS2 antisense eRNA (As-eRNA) siRNA, *TMPRSS2* As-eRNA siRNA with TMPRSS2 (exon1)-ERG (exon4) fusion (T1-ERG) plasmids, or *ERG* siRNAs using lipofectamine (500 cells for each well). After 24 h, cells were fixed and stained by crystal violet. Cells were calculated for each well. Error bars represented mean ± SD for triplicate experiments (n=3). *P* values were shown in the figures.

We previously found that sense eRNAs affected adjacent mRNA expression 42 and in this study we found that antisense eRNA regulated mRNA expression. However, what significance of sense eRNA and antisense eRNA for the transcription of mRNA. To further understand which directional eRNA is more important, we knocked down sense eRNA and/or antisense eRNA in C4-2 cells by LNAs. We screened 8 LNAs for *PSA* antisense eRNA and 9 LNAs for *FKBP5* antisense eRNA, and then chose 2 of them for each antisense eRNA (Figure S5A and S5B). The data showed that mono-KD of sense eRNA or antisense eRNA impaired *PSA* mRNA, and combination KD exhibited more significantly decrease of *PSA* mRNA by LNAs and ASOs (Figure 5A and 5B). We also observed the similar pattern for *FKBP5* mRNA (Figure S5C). In the previous data, we found that ASOs with sense *PSA*-eRNA blocked approximately 40% growth rates in C4-2 cells 15. To identify and compare the antisense eRNA effect in prostate cancer cell growth, we measured the cell viability and colony formation in C4-2 cells by antisense *PSA* eRNA ASOs. The data showed that mono-treatment with ASOs of antisense eRNA downregulated approximated 40% growth rates and 46% colony formation of C4-2 cells (Figure 5C and 5D). Combination treatment with ASOs of sense and antisense *PSA* eRNAs inhibited more than 70% growth rates of cell viability and colony formation (Figure 5C and 5D). In addition, luciferase assay showed that combination treatment with ASOs of sense and antisense *PSA* eRNA blocked 30% more than mono-treatment of each ASO (Figure S5D). Furthermore, the invasion is another important part in cancer progression. TMPRSS2-ERG fusion mutation is over 50% in prostate cancer patients and mediates prostate cancer cell metastasis 60, 61. Thus, we chose the antisense eRNA and mRNA of *TMPRSS2* as study model. In the figure 5E and S5E, we found that knocking-down (KD) of antisense eRNA of *TMPRSS2* downregulated mRNA levels of TMPRSS2-ERG fusion RNAs and invasion capability of VCaP cell. The invasion-decrease of VCaP cells induced by antisense eRNA KD were rescued by overexpression of TMPRSS2-ERG fusion plasmid (Figure 5E). Thus, we concluded that antisense eRNA controlled prostate cancer cell growth and invasion *in vitro*.

**Figure 6:**
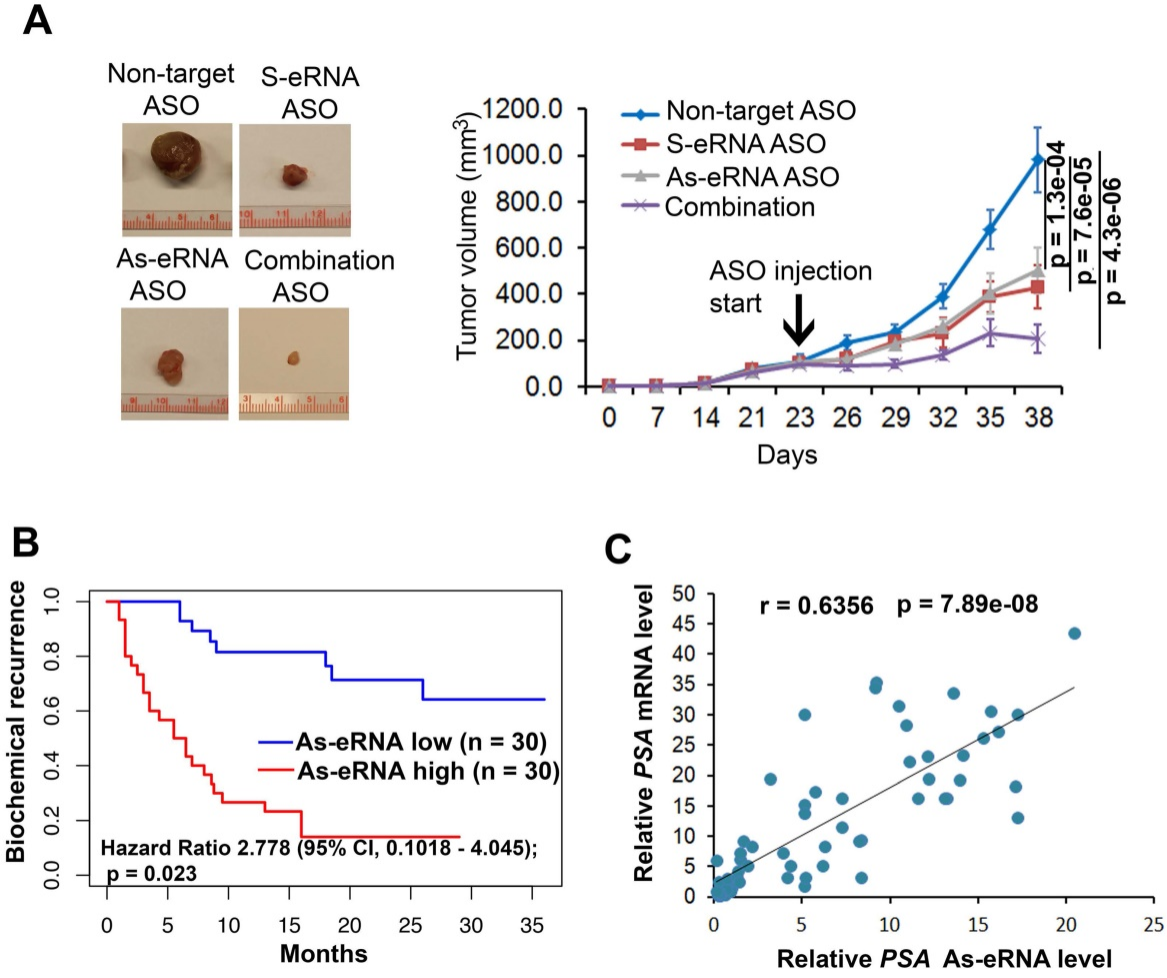
** Antisense eRNA and antisense-enhancer mediate mRNA in mice and in tissues. (A)** Effect of antisense eRNA ASOs on growth of prostate cancer xenografts. 5x10^6^ C4-2 cells were injected into NSG mice (n=6 each group). The tumor growth was measured every 7 days for 42 days, and the data are shown in the bottom panel. Data shown as means ± SD (n=6). Statistical significance was determined by two-tail Student's t-test. *P* values were shown in the figures. **(B)** Kaplan-Meier biochemical recurrence (PSA≥0.2 ng/mL) analysis of the Tianjin Medical University data sets for the relationship between the levels of antisense PSA eRNA, expression of antisense PSA eRNA and biochemical recurrence time in prostate cancers. n=30. Statistical significance is determined by log-rank test. *P* value and hazard ratio were shown in the figures. **(C)** Correlation analysis showing *PSA* mRNA and *PSA* antisense eRNA expression in prostate cancer tissues. Analysis of Tianjin Medical University data sets for levels of *PSA* RNA and *PSA* antisense eRNA were measured by RT-qPCR. n=60. Statistical significance was determined by two-tail Student's t-test.* P* values were shown in the figures.

To further identify the antisense eRNA effect in prostate cancer growth in mice, we treated the mice with ASOs of antisense eRNA by I.P. The results showed that monotherapy with ASOs of antisense eRNA decreased about 30% growth rates of C4-2 cells (Figure 6A). Combination treatment with ASOs of sense and antisense eRNA inhibits more than 60% growth rates of C4-2 xenografts in mice (Figure 6A). Using the cohort in Tianjin Medical University (n=60), we found that high-expressed *PSA* antisense eRNA exhibited shorter biochemical recurrence than patients with low-expressed *PSA* antisense eRNA (Figure 6B). The correlation analysis in the same cohort showed that there was a significantly positive correlation between *PSA* antisense eRNA and mRNA in patients' samples (Figure 6C), suggesting that *PSA* antisense eRNA was positively correlated with clinical marker *PSA'*s level. These data indicated that antisense eRNAs regulated prostate cancer progression in mice and patients' tissues. To further understand the promoter-gene-ending looping significance in tissues, we collected the human single nucleotide polymorphisms (SNP) samples in gene-ending region. Rs2735839 are about 66% people had G/G nucleotides in two allele in *PSA* gene-ending region (same SNP in LNCaP and C4-2), and about 33% A/A nucleotides in the same region 62. It is noticed that patients with AA had more prostate cancer progression and *PSA* serum levels than CC SNP 62-64. We found that AA SNP showed less luciferase activity and promoter-ending interaction than CC SNP using luciferase plasmid in C4-2 cells (Figure S6A). It suggests that patients with CC SNP had more promoter-gene-ending interaction than AA patients. Interestingly, we found that PSA mRNA levels were significantly higher in patients with CC SNP than AA SNP, and PSA antisense ncRNA levels were significantly lower in patients with CC SNP than AA SNP (Figure S6B). These data suggest that both antisense eRNA and promoter-gene-ending looping induced mRNA levels and deduced antisense ncRNA levels of the same locus in prostate cancer cells and tissues.

## Discussion

In human cells, only approximately 20,000 genes (about 2% of the total DNA) are translated into proteins, and more than 90% of genes are transcribed into ncRNAs in the human genome 65. Similarly, it is noted that over 98% of RNAs formed in human cells are ncRNAs 66. eRNAs belongs to ncRNAs and are functional in enhancer-promoter looping and mRNA transcription 5, 15, 54, 58. The eRNAs usually are the RNAs in enhancer with same direction with mRNAs, however antisense eRNAs with opposite direction with mRNAs are rarely reported. Here, we demonstrated that antisense eRNAs were functional ncRNAs. Firstly, we demonstrated that *PSA* antisense eRNA mediated mRNA through inhibiting antisense ncRNA at *PSA* gene locus. Secondly, we found that antisense eRNAs recruited DNMT1 to gene-ending region of *PSA, FKBP5, KLK2* and* TMPRSS2* and increased 5 mC levels in *PSA, FKBP5, KLK2* and* TMPRSS2* gene-ending loci. In mechanism, we found that C-rich region is the key domain for antisense eRNA binding to DNMT1 and antisense eRNA's function. Thirdly, we found a new chromatin looping manner that promoter-gene-ending interaction increased the specific attachment of antisense eRNA targeting to gene-ending region. Fourthly, we found KD of antisense eRNA impaired the cancer cell growth and invasion *in vitro* and cancer progression in tissues (Figures 5 and 6).

Antisense RNAs are transcripts from the opposite direction of sense RNAs. Some of they are small, noncoding, and diffusible molecules, small antisense RNAs included microRNA (miRNA) 21 and NATs 22, 23. They are widespread in eukaryotes and are transcribed in the opposite direction to mRNA, which inhibit mRNA expression through DNA methylation, histone methylation, or dsRNA system 24, 26-29. Through the strand-specific RNA-seq, we found that there were some antisense ncRNA in the gene bodies of *PSA, FKBP5, KLK2* and *TMPRSS2* loci. We also demonstrated that antisense ncRNA of *PSA* inhibited the *PSA* mRNA expression. These data were consistent with reports of antisense ncRNA's function 29, 49, 67, 68. However, the upstream factor of antisense ncRNA remains unclear. The antisense transcripts have been found to originate from independent promoters, shared bi-directional promoters that are situated within genes 16, 21. In this study, we found some enhancers in the gene-ending region with AR, H3K4me1 and DNA methylation ChIP-seq data. We hypothesized that these may be the cis elements of antisense ncRNAs. Interestingly, we demonstrated that mutation or SNP in these regions impair promoter-gene-ending interaction, antisense ncRNA and mRNA levels in prostate cancer cells and human tissues (Figure S6). Furthermore, we found that antisense eRNA involved into this action and recruited DNMT1 in these gene-ending enhancer regions (Figure 2). Taken together, we found a significant link among antisense ncRNA, antisense eRNA and promoter-gene-ending chromatin interaction. It is a new transcript manner for AR target genes.

**Figure 7:**
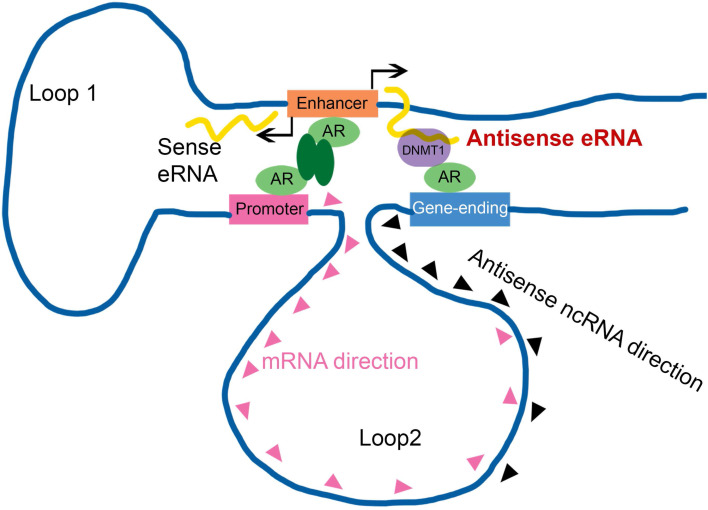
** Antisense eRNA activates mRNA through chromatin double-loop interaction.** A diagram shows double loop model that enhancer interacts with promoter and gene-ending region to facilitate mRNA and deduce antisense ncRNAs.

Enhancer-promoter has been reported for about two decades. It explained how an enhancer can specific activate far distant promoters 3, 8. Some groups already found that one enhancer (or promoter) can interact with multiple regions in genome, including promoters, intragenic and intergenic regions in genome 5, 9, 14, 48. However, people had no idea why promoter interacted with intergenic region. In this study, we found some intergenic region in gene-ending were the new enhancers of antisense ncRNA. Antisense eRNAs recruited DNMT1 there to increased DNA methylation. We analyzed the genome spatial interaction meta-data in published papers 42-44. 4C-seq data and our 3C data showed that the promoters of *PSA, FKBP5, KLK2* and* TMPRSS2* had potential interacted peak in the enhancer region and also in gene-ending region related to promoters (Figure 4A and S4A). However, we found that antisense eRNA mediated mRNA through binding to DNMT1, but not chromatin interaction. Promoter-gene-ending interactions increase the specific of antisense eRNA targeting to gene-ending region (Figure 7). These data can explain how bi-directional eRNAs play different roles to separately and spatially regulate looping and antisense ncRNA/mRNA transcript.

Taken together, we identified AR relating sense eRNAs and antisense eRNAs regulated sense mRNA and antisense ncRNA in prostate cancer cells. Antisense eRNA prevented antisense ncRNA expression in *PSA*, *KLK2*, *FKBP5* and *TMPRSS2* loci, through recruiting DNMT1 on the antisense enhancer and enlarge 5 mC DNA methylation on the gene-ending regions. Importantly, the chromatin exhibited a double looping manner that facilitated sense eRNA to promoter and antisense eRNA to gene-ending region in cis. Collectively, we reported a new interaction that enhancer, promoter and gene-ending region exhibited a spatiotemporally conformation acting mechanism of bi-directional eRNAs. In this regard, the spatiotemporally controlled bi-directional eRNAs were the important factors for allowing them to effectively act at promoter, enhancer and gene-ending region.

## Supplementary Material

Supplementary figure S1-S3.

Supplementary figure S4-S6.

Supplementary table S1.

Supplementary table S2.
