# Supplementary figures and images for: Functional roles of antisense enhancer RNA for promoting prostate cancer progression
Source: Theranostics. 2021 Jan 1;11(4):1780–94. doi: 10.7150/thno.51931 (PMC7778597; doi:10.7150/thno.51931)

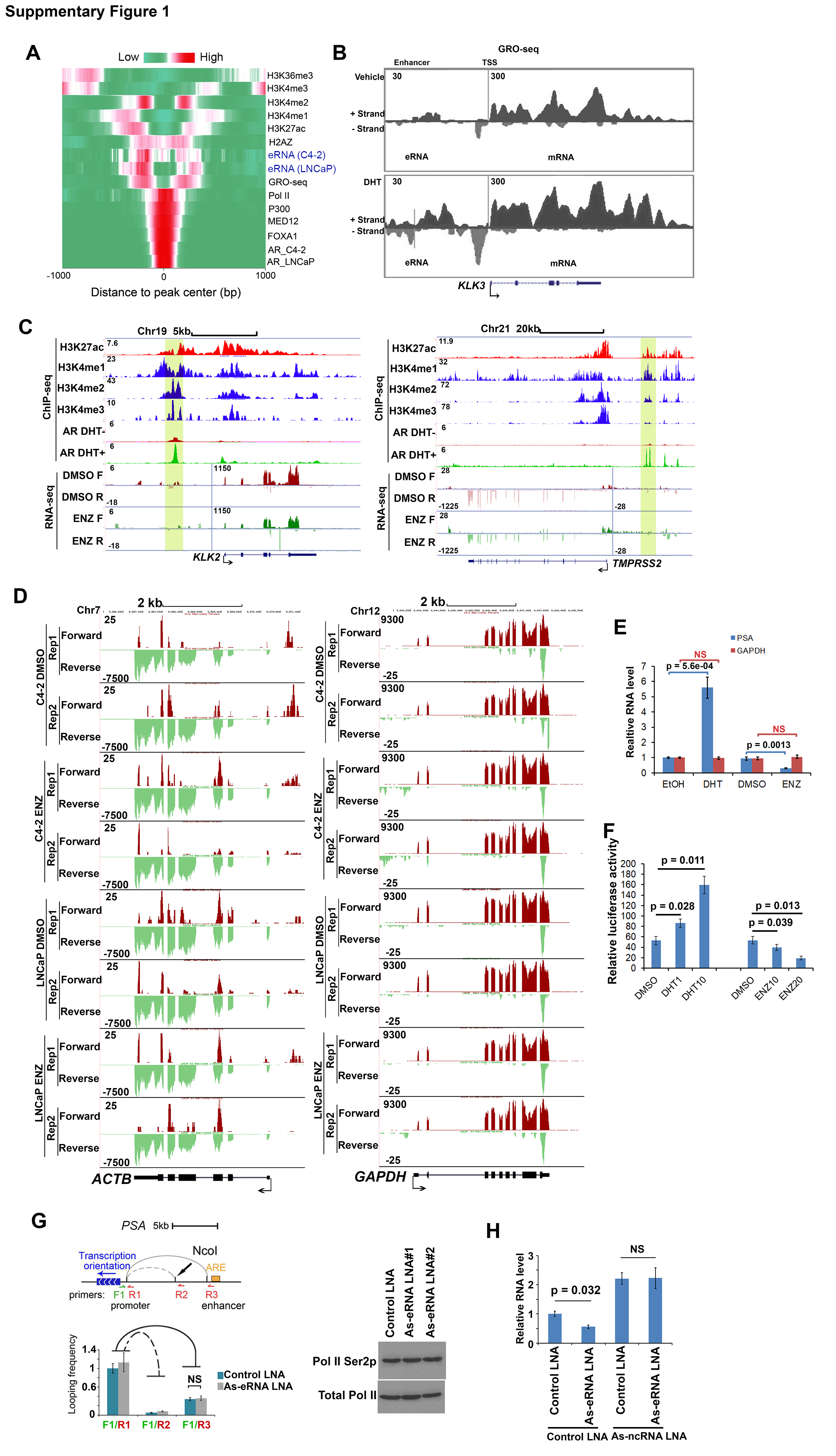

Supplement: Supplementary file 1 — Supplementary figure S1-S3. [file thnov11p1780s1.zip › Supplementatry Figures and Legends1/Figure S1.tif]

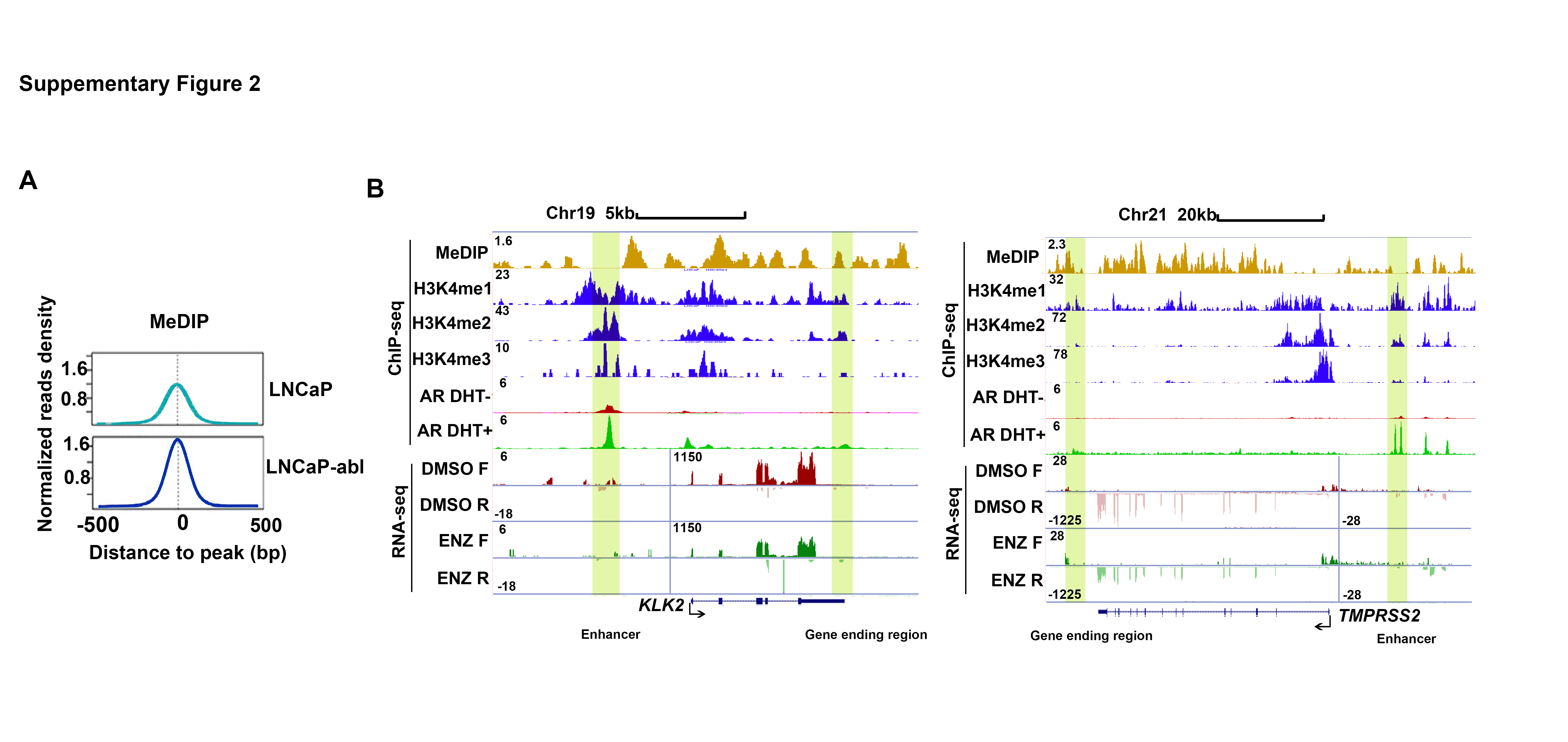

Supplement: Supplementary file 1 — Supplementary figure S1-S3. [file thnov11p1780s1.zip › Supplementatry Figures and Legends1/Figure S2 .tif]

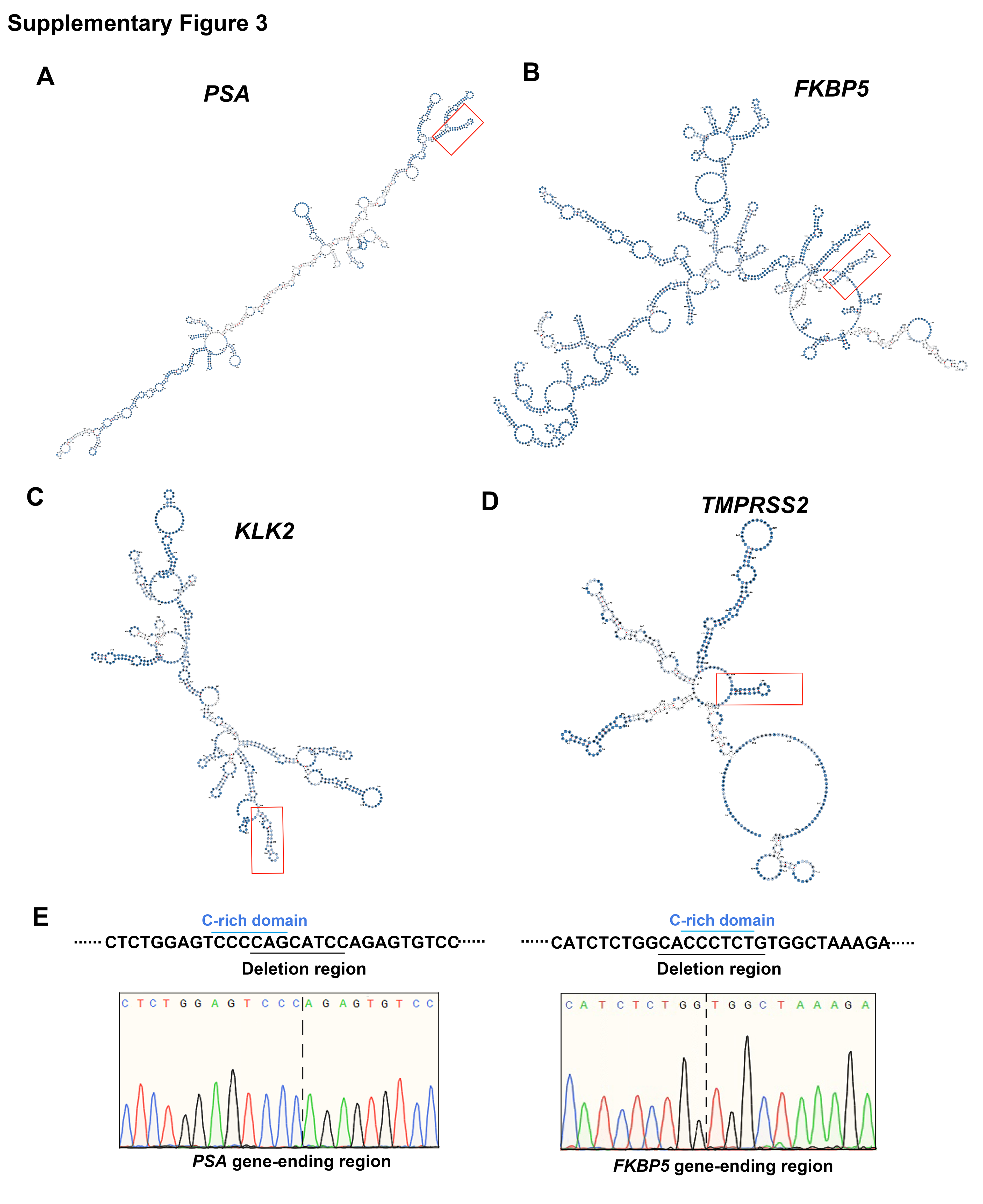

Supplement: Supplementary file 1 — Supplementary figure S1-S3. [file thnov11p1780s1.zip › Supplementatry Figures and Legends1/Figure S3.tif]

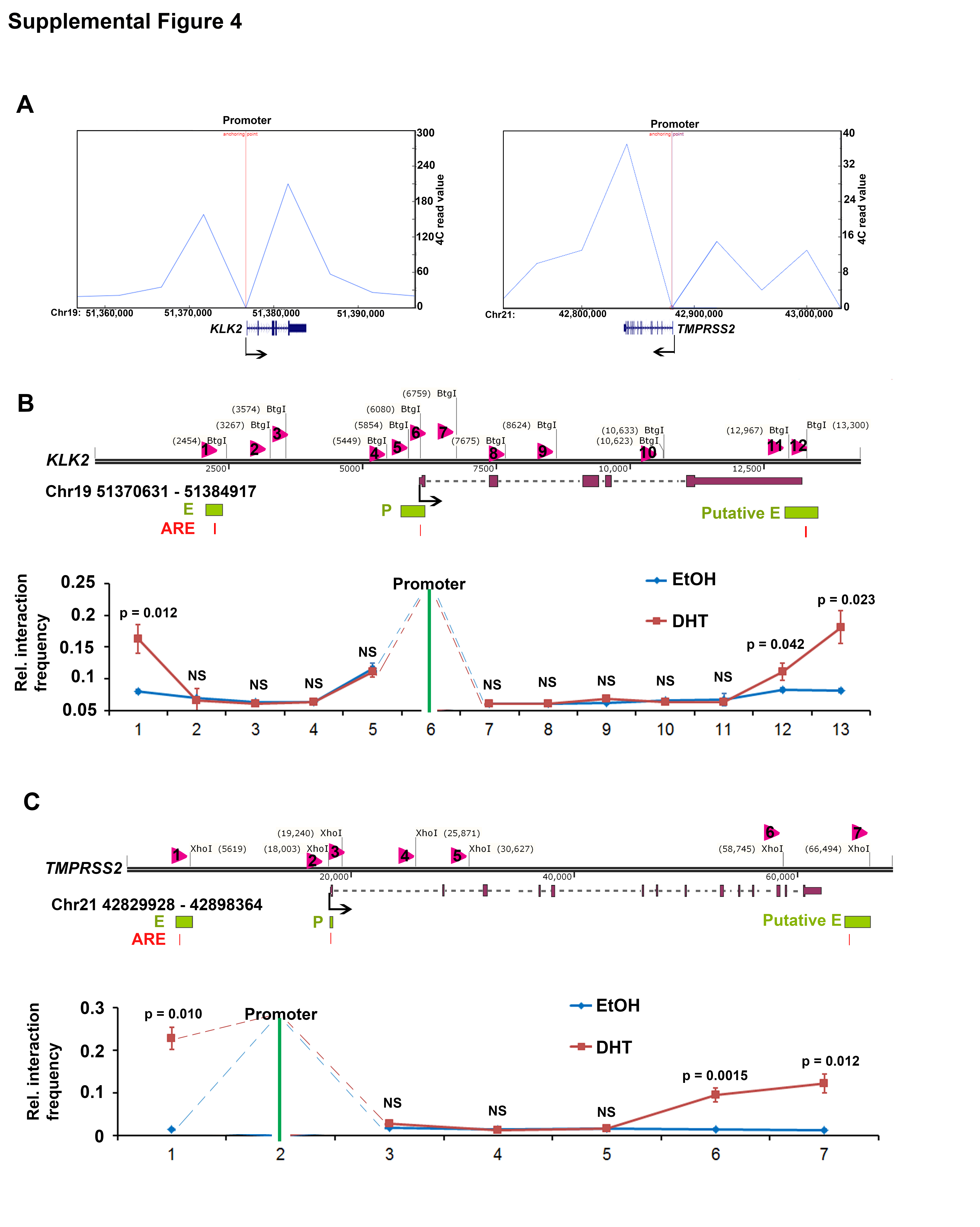

Supplement: Supplementary file 2 — Supplementary figure S4-S6. [file thnov11p1780s2.zip › Supplementatry Figures and Legends2/Figure S4.tif]

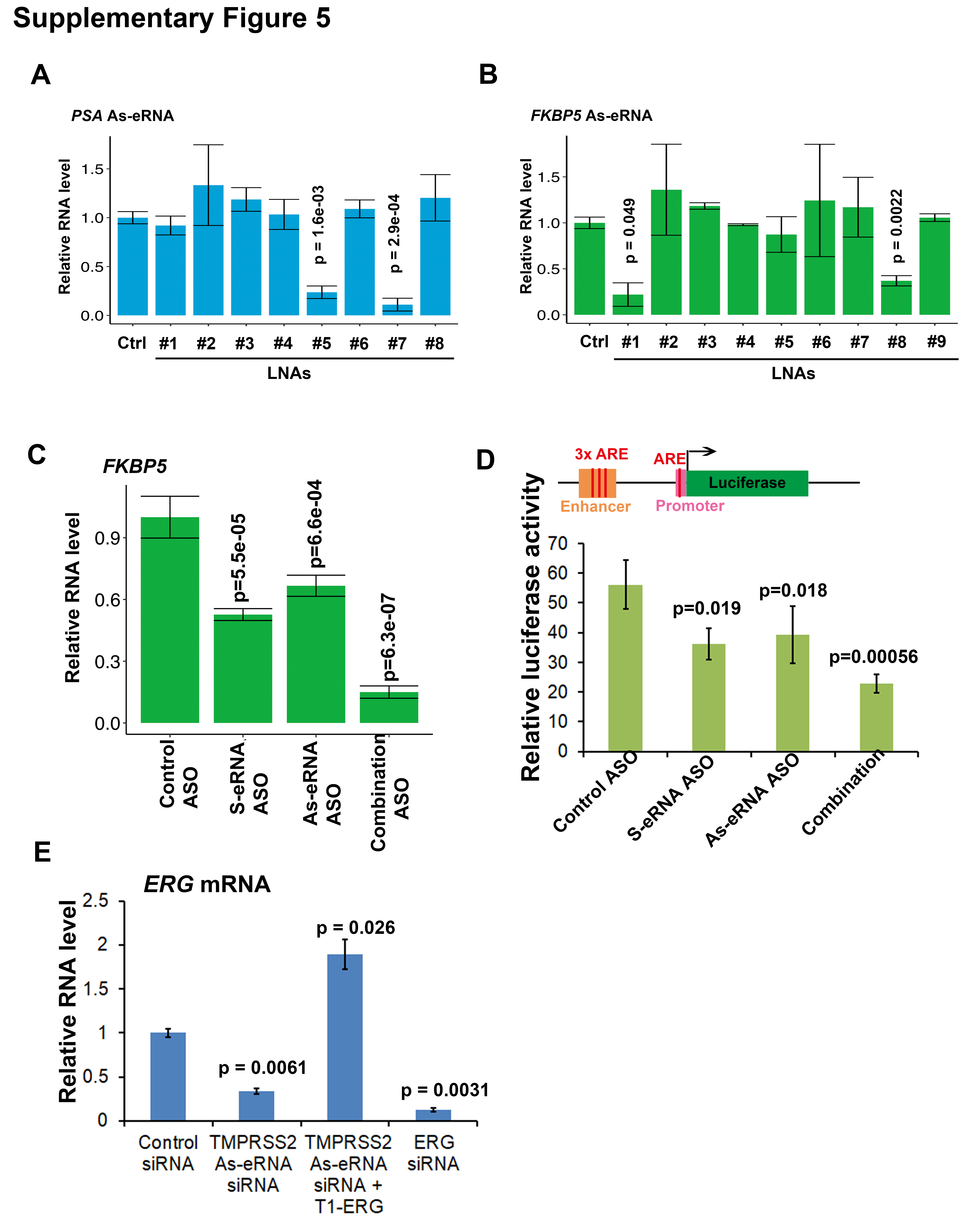

Supplement: Supplementary file 2 — Supplementary figure S4-S6. [file thnov11p1780s2.zip › Supplementatry Figures and Legends2/Figure S5.tif]

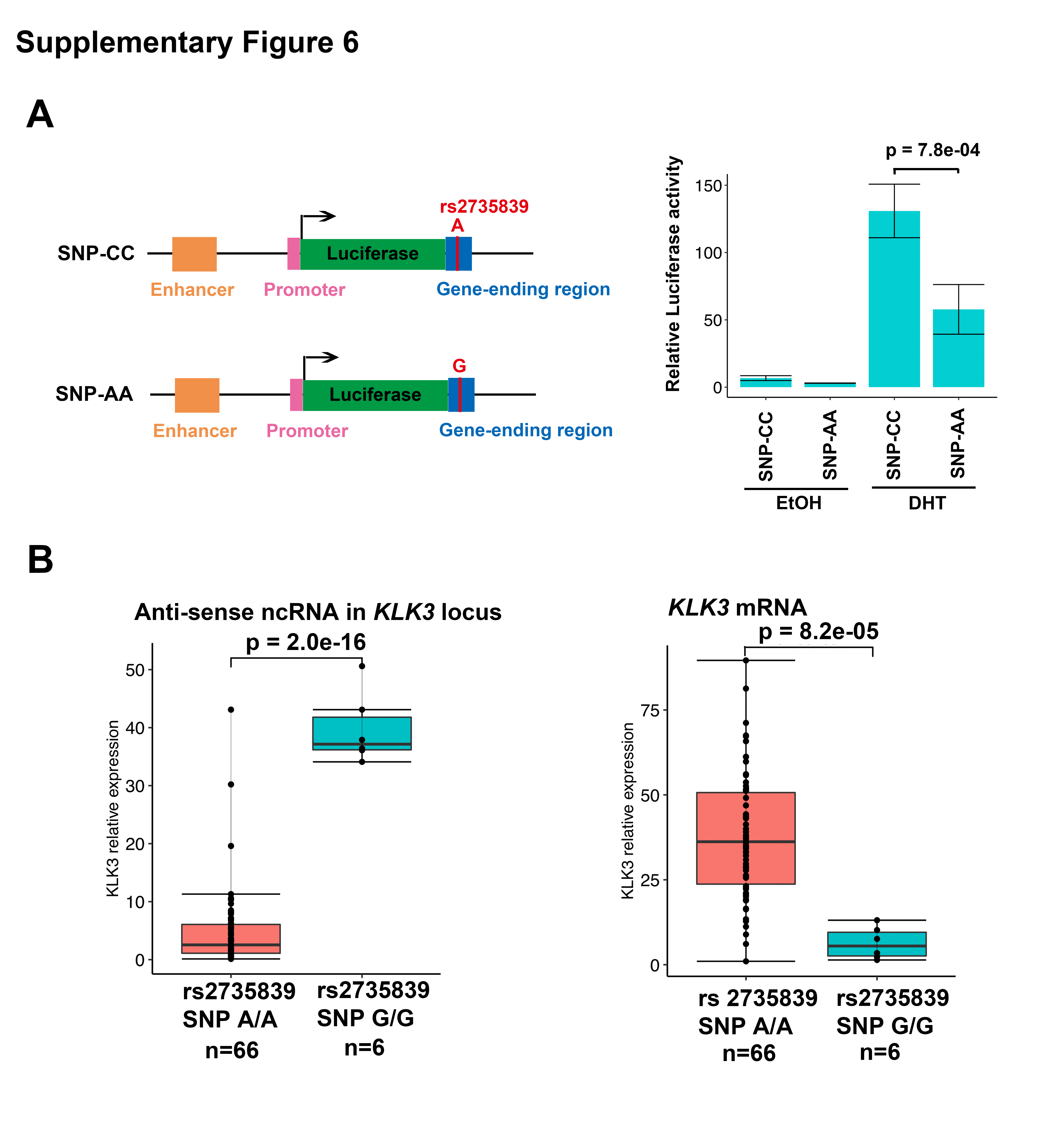

Supplement: Supplementary file 2 — Supplementary figure S4-S6. [file thnov11p1780s2.zip › Supplementatry Figures and Legends2/Figure S6.tif]
