# Supplementary material for: Functional roles of antisense enhancer RNA for promoting prostate cancer progression
Source: Theranostics. 2021 Jan 1;11(4):1780–94. doi: 10.7150/thno.51931 (PMC7778597; doi:10.7150/thno.51931)
Supplement: Supplementary file 2 — Supplementary figure S4-S6. [file thnov11p1780s2.zip › Supplementatry Figures and Legends2/Supplemental Figure Legend.docx]

**Supplemental Figure Legend**

**Supplemental Figure 1. Antisense eRNA is negatively related with antisense mRNA on AR target loci**. Related to Figure 1.

(A) Diagram showed ChIP-seq or RNA-seq signals heatmap on -1000 ~ 1000bp of AR binding site around top 1000 high AR related-eRNA expression.

(B) Screen shots from UCSC genome browser showing signal profiles of eRNA and mRNA expression in LNCaP GRO-seq with or without DHT.

(C) Screen shots from UCSC genome browser showing signal profiles of eRNA and mRNA expression in LNCaP. ChIP-seq in LNCaP and C4-2 are shown as a reference. *KLK2* is in left panel; *TMPRSS2* is in right panel. The enhancer regions are highlighted in yellow box.

(D) Screen shots from UCSC genome browser showing signal profiles of *ACTB* (β-ACTIN) and *GAPDH* mRNA expression in LNCaP and C4-2 with or without ENZ.

(E) mRNA levels of *PSA* and *GAPDH* were measured by qRT-PCR in C4-2 cells with or without DHT or ENZ. Means and standard deviations (error bar) were determined from three replicates. Error bars represent mean ± SD for triplicate experiments. *P* values are shown in the figures. *ACTB* as internal control.

(F) Luciferase activity was measured in C4-2 cells. Renilla activity as internal control. Error bars represent mean ± SD for triplicate experiments. *P* values are shown in the figures.

(G) Evaluation of the enhancer-promoter interaction at *PSA* target gene loci by 3C assays (left panel). Right panel, western blot showed Pol II Ser2 phosphorylation and total Pol II expression in C4-2 cells transfected with control LNA or antisense LNAs.

(H) mRNA levels of *PSA* were measured by qRT-PCR in C4-2 cells knocked down with antisense eRNA LNAs. Means and standard deviations (error bar) were determined from three replicates. Error bars represent mean ± SD for triplicate experiments. *P* values are shown in the figures. *GAPDH* as internal control.

**Supplemental Figure 2. DNA methylation in antisense promoter is critical for expression of antisense ncRNA.**

(A) Diagram showed MeDIP-seq signals on -500 ~ 500bp of gene-ending site around top 100 high AR related-eRNA expression.

(B) Screen shots from UCSC genome browser showing signal profiles of eRNA and mRNA expression in LNCaP and C4-2. MeDIP-seq in Abl cells are shown as a reference. *KLK2* is in left panel; *TMPRSS2* is in right panel. The enhancer regions are highlighted in yellow box.

**Supplemental Figure 3. Secondary structure of antisense eRNAs.**

(A-D) Secondary structure of *PSA, FKBP5, KLK2 and TMPRSS2* predicted by <https://rna.tbi.univie.ac.at>. C-rich domains were showed by red boxes.

(E) Sanger sequence for CRISPR deletion cell lines in *PSA* and *FKBP5* gene-ending regions.

**Supplemental Figure 4. Antisense eRNA's function in cis relies on new two-looping interaction.**

(A) Virtual circularized chromosome conformation capture (4C)-seq data from <http://promoter.bx.psu.edu/hi-c/chiapet.php> showing potential signal binding regions with *KLK2 and TMPRSS2* promoter in NHEK GM12878 and LNCaP cells.

(B and C) The diagram showing that chromosome conformation capture (3C) assay with BtgI digesting in *KLK2* locus and XhoI digesting in *TMPRSS2* locus. Evaluation of the enhancer-promoter-antisense enhancer (gene-ending region) interaction at *FKBP5 and PSA* target gene loci by 3C assays.

**Supplemental Figure 5. Antisense eRNA and antisense-enhancer mediate mRNA in cells.**

(A - C) The expressions of *PSA and FKBP5* are measured by qRT-PCR in C4-2 cells transfected by LNAs. Means and standard deviations (error bar) were determined from three replicates. Error bars represent mean ± SD for triplicate experiments. *P* values are shown in the figures. *GAPDH* as internal control.

(D) Upper panel, diagrams showed Luciferase plasmids with enhancer and promoter of AR recognized elements (ARE). low panel, luciferase activity was measured in C4-2 cells. Renilla activity as internal control.

(E) The expressions of *TMPRSS2* are measured by qRT-PCR in C4-2 cells transfected by control siRNA, TMPRSS2 as-eRNA siRNA with or without TMPRSS2-ERG fusion expression plasmids or ERG siRNA. Means and standard deviations (error bar) were determined from three replicates. Error bars represent mean ± SD for triplicate experiments. *P* values are shown in the figures. *GAPDH* as internal control.

**Supplemental Figure 6. Antisense eRNA and antisense-enhancer mediate mRNA in tissues.**

(A) Left panel, diagrams showed Luciferase plasmids with enhancer, promoter and gene-ending regions of PSA. Right panel, luciferase activity was measured in C4-2 cells. Renilla activity as internal control.

(B) Box and whisker plot showing *PSA* RNA signals expressed in prostate cancer tissues. Analysis of Tianjin Medical University data sets for levels of *PSA* RNA were distinguished SNP: GG and AA. n=66 for A/A group, n=6 for G/G group.
